# Microstructural evidence for crystallization regimes in mafic intrusions: a case study from the Little Minch Sill Complex, Scotland

**DOI:** 10.1007/s00410-018-1525-7

**Published:** 2018-11-09

**Authors:** Gautier Nicoli, Marian Holness, Jerome Neufeld, Robert Farr

**Affiliations:** 1 Department of Earth Sciences, University of Cambridge, Downing Street, CB2 3EQ Cambridge, UK; 2 BP Institute for Multiphase Flow, University of Cambridge, Madingley Road, CB3 0EZ Cambridge, UK; 3 Department of Applied Mathematics and Theoretical Physics, University of Cambridge, Wilberforce Rd, CB3 0WA Cambridge, UK; 4 Jacobs Douwe Egberts, Ruscote Ave, OX16 2QU Banbury, UK

**Keywords:** Sill, Crystallization, Convection, Microstructure, Olivine, Grain size

## Abstract

The magma forming the 20 m thick crinanitic/picrodoleritic Dun Raisburgh sill, part of the Little Minch Sill Complex of NW Scotland, comprised a mafic carrier liquid with a crystal cargo of plagioclase and olivine (1 vol%). The olivine component of the cargo settled on the floor of the intrusion while the more buoyant plagioclase component remained suspended during solidification, resulting in a relatively high plagioclase content in the centre of the sill. The settled olivine grains form a lower fining-upwards sequence overlain by a poorly sorted accumulation formed of grains that grew within the convecting magma. The accumulation of olivine on the sill floor occurred over 5–10 weeks, synchronous with the upwards-propagation of a solidification front comprising a porous (~ 70 vol% interstitial liquid) plagioclase-rich crystal mush.

## Introduction

The processes controlling the crystallization of magmatic intrusions in the Earth’s crust depend on the physical behaviour of particle-bearing magmas. From early emplacement to complete solidification, the fluid dynamic behaviour of the evolving magmatic system defines the internal architecture of the intrusion (Egorova and Latypov 2013; Holness et al. 2017a). This behaviour is the consequence of the interplay between intrusion size, the temperature of the host rocks (e.g. Irvine 1970; Annen 2011) and magma chemistry (e.g. Mysen 1982; Sparks and Huppert 1984). During, and shortly after, emplacement, the magnitude of the crystal load is also important (e.g. Gibb and Henderson 2006; Marsh 2013; Holness et al. 2017a). Recent work, building on the pioneering advances of Bowen (1915), has shown how fluid dynamical behaviour during crystallisation can be deduced from the information retained in the microstructural features preserved in fully solidified intrusions (Holness et al. 2017a, b). In the present contribution, we present the results of a detailed investigation of an olivine-phyric sill from the Little Minch Sill Complex, exposed on the Trotternish Peninsula, Isle of Skye, Scotland (first described by Gibb and Gibson 1989), and show that a combination of petrographic observations and thermodynamic modelling can be used to decode fluid dynamic behaviour during crystallization.

## Previous work

The work of Holness et al. (2017a) was focussed on the Shiant Isles Main Sill, a composite member of the Little Minch Sill Complex. The Shiant Isles Main Sill comprises four separate intrusions (Gibb and Henderson 1996), of which the larger two consist of a 24 m early picrite intrusion and a later 135 m picrodolerite-crinanite unit (PCU). The picrodoleritic basal part of the PCU comprises an ~ 50 m accumulation of settled olivine grains, in which the average grain size decreases upwards in the lowermost 10 m but then increases upwards in the uppermost ~ 40 m. Holness et al. (2017a) argued, on the basis of stratigraphic variations in both bulk-rock Cr compositions and the extent of clustering of olivine grains, that olivine accumulated on the floor of the PCU in two stages: the first of which occurred by the settling of most of the olivine cargo from the incoming magma, whereas the later coarsening-upwards sequence resulted from a slower progressive accumulation of olivine grains and clusters from the convecting magma.

These findings suggest that one of the key ways of interrogating the fluid dynamic regime during crystallisation of tabular magmatic intrusions and lava flows is via a close examination of the accumulated crystal cargo on the floor (e.g. Drever 1952; Arndt 1986; Hoshide et al. 2006). In systems in which there is negligible crystal growth, the accumulation formed through the settling of a polydisperse crystal load fines upwards (Mulder and Alexander 2001): fining-upwards accumulations are common in sedimentary rocks. In detail, however, the structure of a fining-upwards sequence can be used to differentiate between gravitational settling from a static magma and gravitational settling from a convecting magma (Holness et al. 2017a). The fining-upwards sequence formed by settling from a static magma is characterised by the complete disappearance of progressively smaller size classes upwards in the accumulation: this is known as coarse-tail grading (e.g. Palladino and Valentine 1995; Postma et al. 2009). In contrast, a basal accumulation formed in a system in which the liquid is vigorously convecting is characterized by a gradual upwards phasing-out of each class size (Holness et al. 2017a). In a convecting magmatic system, the particle size distribution is likely to be further affected by synneusis, as crystals suspended in the convecting magma come into contact with each other to form clusters that progressively become larger the longer they remain suspended (e.g. Schwindinger and Anderson 1989). Consequently, the settling speed of the aggregating particles increases (Schwindinger 1999).

Holness et al. (2017a) argued that if convection in the intrusion is maintained as a consequence of non-symmetrical heat loss (e.g. if the intrusion is underlain by an earlier, still hot, intrusion), some of the crystal cargo might remain in suspension for considerable periods, resulting in significant amounts of both synneusis and further growth of the suspended crystals in the progressively cooling magma. Settling of these crystals then results in the formation of a coarsening-upwards sequence overlying the earlier fining-upwards accumulation.

## Geological setting

**Fig. 1:**
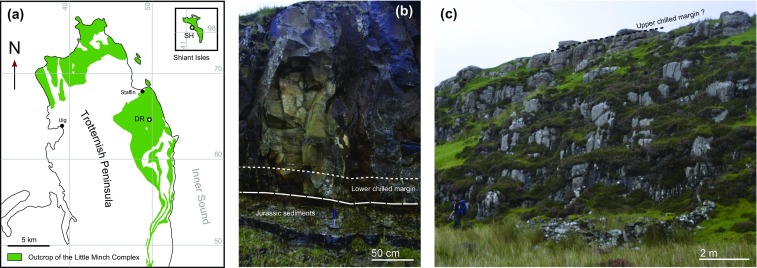
**a** Map of the Trotternish Peninsula, northern part of the Isle of Skye Scotland, and the Shiant Isles. DR indicates the location of the Dun Raisburgh sill; **b** base of the sill and contact with the Jurassic sedimentary country rock; **c** upper exposed part of the sill. The summit of the cliff is likely to be close to the upper margin, although the actual contact with the country rock is not preserved

The *ca*. 60 Ma Little Minch Sill Complex is mainly located offshore along the north-west coast of Scotland (Gibb and Gibson 1989). On land, the sills intrude Jurassic sediments and shape the landscape of the northern part of the Isle of Skye (Trotternish Peninsula) and the Shiant Isles (Fig. 1). The sills are mafic and many are composite (Gibson 1988, 1990; Gibb and Henderson 1996; Holness et al. 2017a). Attempts have been made to correlate the different outcrop localities using mineral modes, whole-rock major and trace element compositions, and Sr isotopes (Gibson 1988, 1990; Gibb 1992). The sills comprise three lithologies: picrite (olivine > 40%, plagioclase, clinopyroxene); picrodolerite (olivine 10–20%, plagioclase, clinopyroxene) and crinanite (olivine < 5%, plagioclase, clinopyroxene, analcime). The thickness of the sills varies from ~ 20 to > 100 m.

In this study, we focus on the crinanitic/picrodoleritic, olivine-phyric, Dun Raisburgh sill (Gibson and Jones 1991) (Fig. 1). At our sampling site on the east coast of the Trotternish Peninsula (57°36′4.15″N; 6°10′55.30″W) (Fig. 1), this sill is at least 18.8 m thick and intrudes into Jurassic sediments. As described by Gibson and Jones (1991), there is no evidence for internal chilled margins: the Dun Raisburgh sill is therefore likely to have formed from a single injection of basaltic magma.

Gibb and Henderson (1996) showed that the magma forming the Little Minch Sill Complex comprised a carrier liquid with a crystal load mainly of olivine with minor plagioclase. The lower contact of the intrusion, chilled against the sedimentary country rocks, is well-exposed. In common with many of the Trotternish sills (Henderson et al. 2000), much of the uppermost part of the sill has been eroded: we found no evidence of an upper contact in the field at our sampling site. However, microstructural observations (see below) suggest that the total sill thickness does not exceed 20 m. We collected 24 samples along a vertical transect.

## Petrography

### The lower chilled margin of the Dun Raisburgh Sill

**Fig. 2:**
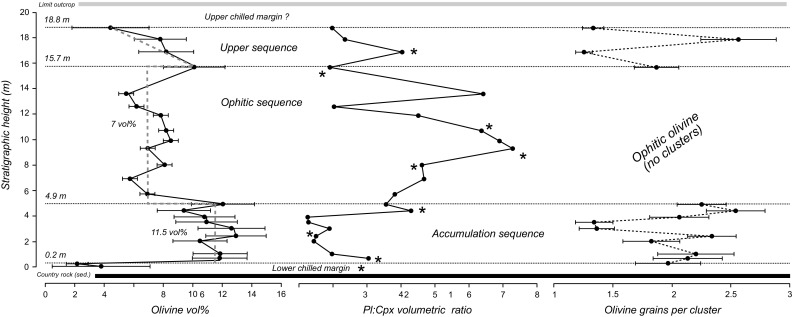
Stratigraphic variation of the olivine mode (vol%) together with the plagioclase/clinopyroxene (Pl/Cpx) volumetric ratio (both determined by point counting) and the number of olivine grains per cluster (with isolated olivine grains counted as one grain per cluster) in the Dun Raisburgh sill. The horizontal dashed lines show the boundaries between the different stratigraphic subdivisions of the sill (see text). The asterisks indicate the stratigraphic position of our thin sections containing stellate plagioclase clusters. The olivine mode in the accumulation and upper sequences was determined by point counting, with the associated uncertainties calculated according to Van der Plas and Tobi (1965). The olivine mode in the ophitic sequence was estimated from image analyses using ImageJ software (Schneider et al. 2012)

**Fig. 3:**
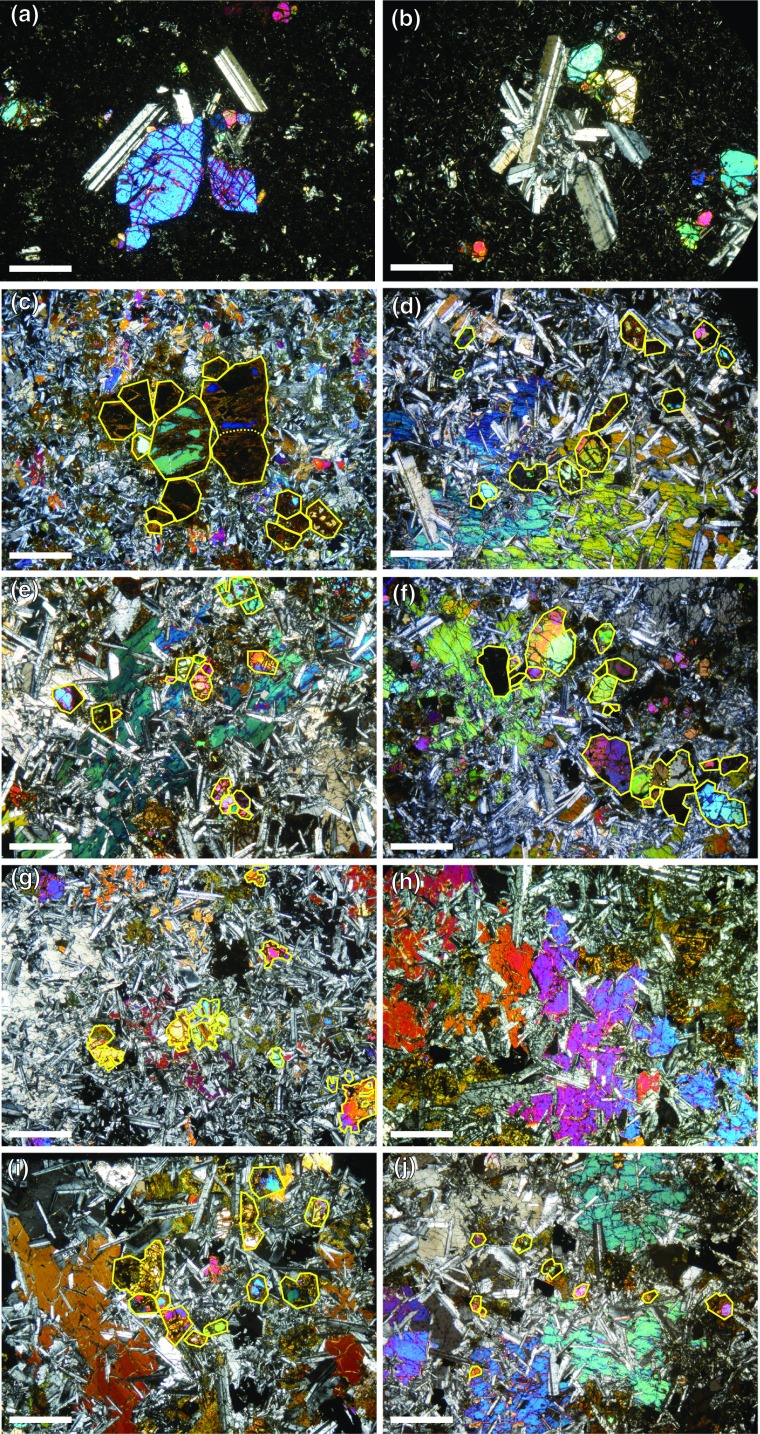
Photomicrographs of the Dun Raisburgh sill under crossed polarised light, showing the microstructural evolution from the base to the top of the intrusion. The scale bar in all images is 1 mm long. We have outlined a few representative clusters to illustrate the change in particle size between the different stratigraphic heights. **a, b** Sample RB10 (0 m stratigraphic height). Well–facetted olivine and plagioclase clusters from the original crystal cargo in the lower chilled margin; **c** bottom (sample RB12, 0.65 m stratigraphic height) and **d** top (sample RB19, 3 m stratigraphic height) of the fining-upwards sequence. The olivine phenocrysts form clusters which decrease in size towards the top of the fining-upwards sequence. The augite forms relatively small interstitial crystals within a plagioclase-dominated matrix; **e** bottom (sample RB16, 3.9 m stratigraphic height) and **f** top (sample RB27, 4.9 m stratigraphic height) of the poorly-defined coarsening-upwards sequence. The olivine clusters comprise a similar amount of grains within this part of the sill (Fig. 2); **g, h** sample RB05 (10.7 m stratigraphic height), from the ophitic olivine region of the sill. The augite (**h**) forms extensive interstitial oikocrysts enclosing plagioclase; **i** bottom (sample RB02, 15.7 m stratigraphic height) and **j** top (sample RB01, 17.9 m stratigraphic height) of upper sequence at the top of the sill. The olivine phenocrysts appear to form large clusters at the bottom of this sequence. Augite is significantly finer-grained than that in the underlying ophitic sequence (Fig. 4)

Samples from the lower chilled margin (RB10 and RB17, see Table 1) contain olivine and plagioclase phenocrysts. These components, derived from the crystal cargo, comprise ~ 2.5 vol% and ~ 5.5 vol%, respectively, of the chill (Fig. 2). While chilled margins may not be fully representative of the original magma composition, in particular the volumetric proportion of the crystal load (Egorova and Latypov 2013), the presence of olivine and plagioclase phenocrysts in the chill shows that the cargo contained these minerals. The olivine phenocrysts are generally euhedral and equant (Fig. 3a), and occur as isolated single grains and as sintered clusters. By counting the isolated grains as clusters containing only one grain, we calculate the average number of grains per cluster (as viewed in thin section) as ~ 2 (Fig. 2). In common with the lower margin of the PCU of the Shiant Isles Main Sill (Holness et al. 2017a), grains within a single (poly-crystalline) cluster commonly display similar optical orientations and planar grain boundaries parallel to growth faces of euhedral olivine, consistent with cluster formation by synneusis (Schwindinger and Anderson 1989). Plagioclase phenocrysts are generally isolated, although rare stellate clusters are also present (Fig. 3b). Some grain clusters contain both olivine and plagioclase (Fig. 3a, b): such poly-phase clusters may indicate an origin in a disaggregated troctolitic crystal mush.

**Table 1:** Microstructural data for olivine, plagioclase and clinopyroxene in the Dun Raisburgh Sill

Sample	(m)	Mode (vol%)			Olivine particle size (mm)										Plagioclase				
		Ol	*σ*	Pl:Cpx	n_Ol_	${D_{{\text{4}},{\text{3}}}}$	${D_{{\text{3}},{\text{2}}}}$	${D_{{\text{3,1}}}}$	σ_est_	e_est_	δD	$D{*_{{\text{4}},{\text{3}}}}$	PPC	σ		n_Pl_	AR	2σ	log(year)
Lower Chilled Margin																			
RB10	0	3.8	3.3	–	33	1.53	1.09	0.90	0.58	0.48	–	–	–	–		167	3.42	0.14	–3.20
RB17	0.2	2.2	0.7	–	385	0.82	0.65	0.59	0.49	0.09	–	–	1.97	0.28		–	–	–	–
Fining-upwards Sequence																			
RB12	0.65	11.8	1.9	3.1	303	1.24	0.93	0.85	0.54	0.13	0.17	1.07 ± 0.18	2.13	0.29		189	3.08	0.14	− 1.58
RB13	1	11.9	1.8	2.0	310	1.12	0.90	0.82	0.47	0.09	0.17	0.96 ± 0.16	2.20	0.32		156	2.97	0.17	− 1.21
RB14	2	10.5	1.8	1.4	278	0.96	0.84	0.80	0.36	0.05	0.15	0.80 ± 0.12	1.83	0.24		152	3.64	0.26	− 0.62
RB15	2.4	12.9	2.0	1.5	268	1.01	0.87	0.82	0.38	0.06	0.16	0.85 ± 0.14	2.34	0.21		151	3.61	0.22	− 0.47
RB19	3	12.6	2.3	1.9	214	0.80	0.73	0.71	0.30	0.05	0.13	0.67 ± 0.09	1.36	0.15		167	4.13	0.28	− 0.29
RB20	3.5	10.9	2.1	1.3	224	0.72	0.66	0.65	0.30	0.04	0.12	0.60 ± 0.07	1.34	0.16		183	3.89	0.24	− 0.17
Coarsening-upwards Sequence																			
RB16	3.9	10.8	2.1	1.3	226	0.96	0.83	0.79	0.37	0.06	0.15	0.81 ± 0.12	2.06	0.25		160	4.25	0.31	− 0.08
RB09	4.4	9.4	1.8	4.3	261	1.05	0.92	0.86	0.36	0.06	0.17	0.88 ± 0.15	2.54	0.25		150	3.54	0.22	0.00
RB27	4.9	12.0	2.1	3.6	231	1.07	0.81	0.71	0.53	0.14	0.15	0.92 ± 0.14	2.25	0.21		140	3.47	0.20	0.08
Ophitic Sequence																			
RB08	5.7	6.8	0.5	3.8	–	–	–	–	–	–	–	–	–	–		167	3.39	0.18	0.17
RB26	6.9	5.9	0.5	4.7	–	–	–	–	–	–	–	–	–	–		166	3.47	0.20	0.27
RB07	8	8.1	0.5	4.6	–	–	–	–	–	–	–	–	–	–		170	3.30	0.22	0.33
RB06	9.3	6.9	0.5	7.3	–	–	–	–	–	–	–	–	–	–		165	3.40	0.27	0.35
RB24	9.9	8.5	0.5	6.9	–	–	–	–	–	–	–	–	–	–		152	3.66	0.25	0.35
RB05	10.7	8.2	0.5	6.4	–	–	–	–	–	–	–	–	–	–		126	3.06	0.17	0.34
RB23	11.9	7.8	0.5	4.5	–	–	–	–	–	–	–	–	–	–		163	3.59	0.21	0.28
RB04	12.6	6.2	0.5	1.9	–	–	–	–	–	–	–	–	–	–		188	3.62	0.25	0.23
RB03	13.6	5.5	0.5	6.4	–	–	–	–	–	–	–	–	–	–		170	3.88	0.33	0.13
Upper sequence																			
RB02	15.7	10.1	2.1	1.9	209	0.94	0.85	0.82	0.31	0.05	0.25	0.68 ± 0.17	1.87	0.19		155	4.12	0.27	− 0.23
RB21	16.9	8.2	1.9	4.0	217	0.77	0.74	0.74	0.20	0.03	0.22	0.55 ± 0.12	1.26	0.07		160	3.88	0.26	− 1.21
RB01	17.9	7.8	1.8	2.4	231	0.89	0.76	0.71	0.39	0.07	0.23	0.66 ± 0.15	2.57	0.32		221	3.68	0.19	− 0.62
RB00	18.8	4.4	2.6	2.0	62	0.56	0.55	0.55	0.16	0.04	–	–	1.33	0.09		154	3.06	0.17	− 3.20
Upper Chilled Margin																			

*nOl, nPl* number of analysed grains, *PPC* average particle per cluster, *AR* avergae plagioclase aspect ratio, D*_4,3_ corrected particle size from overgrowth

**Fig. 4:**
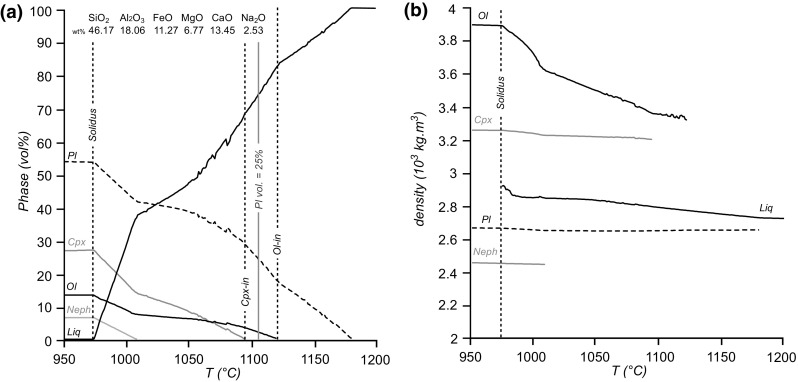
Calculated evolution of the carrier liquid during cooling at 1 kbar (see text for description and method). **a** Volume (%) of the different phases precipitating from the carrier liquid during cooling at 1 kbar. The dotted vertical line indicates the solidus at 975 °C. The liquid saturates in Cpx at 1095 °C and in Ol at 1120 °C. The plagioclase mode reaches 25 vol% at 1105 °C (at which point the olivine mode is 2.4 vol%). **b** The variation of density of the different phases as a function of temperature

### Stratigraphic subdivisions of the Dun Raisburgh sill

The non-chilled parts of the Dun Raisburgh sill comprise plagioclase, clinopyroxene (augite) and olivine, with minor amounts of interstitial magnetite, analcime, apatite and zeolite (Gibb and Henderson 1996). We subdivide the stratigraphy of the Dun Raisburgh sill into three, on the basis of variations in olivine mode, the volumetric plagioclase to clinopyroxene (Pl/Cpx) ratio, olivine habit and the extent of clustering of the olivine (Fig. 2).The lowermost subdivision, the accumulation sequence, comprises the basal 4.9 m (defined by 9 samples), overlain by the central subdivision, the ophitic sequence, (4.9–15.7 m; samples RB08-RB03), with the uppermost subdivision, the upper sequence, (defined by 4 samples) comprising the remaining ~ 3 m of the stratigraphy.

Clinopyroxene (augite) is interstitial throughout the sill. In most of the sill, plagioclase is euhedral and randomly oriented. The plagioclase component of the crystal cargo, which is clearly identifiable in the chilled margin, is not easy to identify in the central part of the sill, with the exception of the stellate clusters which are randomly distributed throughout the stratigraphy (Fig. 2) (c.f. Gibb and Henderson 1996, 2006).

The accumulation sequence is comparatively olivine-rich with an average mode of 11.5 ± 0.9 vol%. Olivine forms well-defined euhedral grains that appear either isolated or in clusters (as viewed in thin section) (Fig. 3c-d). The number of olivine grains per cluster (as sampled by our randomly-oriented thin sections) increases upwards from 1.3 to 2.3. The average Pl/Cpx volumetric ratio in the first 3 m is 2.0 ± 0.6. The ratio then increases to ~ 3.0 ± 0.4 at the top of the accumulation sequence.

The ophitic sequence in the central region of the sill is characterised by interstitial olivine (Fig. 3g), with an average olivine mode of 7.0 ± 1.0 vol%. Towards the centre of the sill, ophitic clinopyroxene is chemically zoned (detectable by variation in birefringence) supportive of in situ fractionation during crystallisation of the interstitial liquid in a crystal mush (Fig. 3h). The Pl/CPx volumetric ratio describes a reverse S-shape with maximum values concentrated above or near the middle of the intrusion (9.3 m stratigraphic height) (Fig. 2).

The upper sequence is characterized by the re-appearance of euhedral olivine and a decrease in olivine mode from 10 to 4 vol%, an average Pl/Cpx volumetric ratio of 2.5 ± 0.9 and an average of 1.9 olivine grains per cluster. The interstitial augite comprises a poikilitic core containing abundant fine-grained plagioclase and an outer plagioclase-poor rim (Fig. 3i, j).

### The upwards-propagating solidification front

The accumulation sequence in the Dun Raisburgh sill is relatively olivine-poor, at ~ 11.5 vol%, compared to that in the PCU of the Shiant Isles Main Sill which contains up to 55 vol% olivine (Holness et al. 2017a). Thus, whereas the olivine mode in the Shiant Isles Main Sill PCU is sufficiently high that olivine alone formed a mechanically coherent framework (Dong et al. 2006; Yang et al. 2007), there was insufficient olivine at the base of the Dun Raisburgh sill to do so. The olivine on the floor of the Dun Raisburgh sill therefore must have accumulated synchronously with the accumulation, or in situ nucleation and growth, of phases other than olivine. The Dun Raisburgh olivine accumulation sequence must have formed while the sill was solidifying, with the olivine cargo settling onto a continuously thickening, mechanically-coherent mush made of other minerals. Since augite is interstitial throughout the sill, the mineral dominating the crystal mush in these early stages must have been plagioclase.

To estimate the porosity of the upwards-propagating plagioclase-dominated mush, and evolution of modal mineralogy during solidification, we modelled the cooling of the parental magma composition at 1 kbar (Fig. 4a), using the PicrPL liquid composition of Gibbs & Henderson (2006), version 6.7.5 of Perple_X (Connolly 2009) and the solution models for mafic and ultramafic magmas of Jennings and Holland (2015) in a NCFMAS system. Known causes of uncertainty in our modelling include the absence of analcime, because the absence of H_2_O in the thermodynamic dataset means that nepheline is predicted to crystallise instead. Additionally, the absence of Fe^3+^ in our model precludes the formation of magnetite.

Our calculations suggest that plagioclase is the first phase to crystallise from the PicrPL liquid composition at 1180 °C, followed by olivine (Ol) at 1120 °C, clinopyroxene at 1095 °C, and nepheline (Ne) at 1010 °C. The presence of both plagioclase and olivine phenocrysts in the chilled margin suggests that the temperature of the incoming magma was between 1120 °C and 1095 °C. Phase proportions in the completely solidified rock (which occurs at 975 °C) are Pl: 53.28 vol%; Cpx: 26.78 vol% (Pl/Cpx = 2); Ol: 13.26 vol% and Ne: 6.50 vol% (Fig. 4a).

The Pl/Cpx volumetric ratios in the Dun Raisburgh sill are, within error, only close to the predicted value of 2.0 ± 0.6 in the accumulation sequence, and are higher than this value for most of the rest of the stratigraphy. This suggests that the accumulation sequence did not contain any of the plagioclase component of the magma’s crystal cargo, which was instead concentrated into the overlying parts of the sill. This is consistent with the buoyant nature of the plagioclase component of the cargo (Fig. 4b) which, for much of the lifetime of the sill, must have remained suspended in the magma.

In contrast, the Pl/Cpx ratio is close to the predicted value in much of the PCU of Shiant Isles Main Sill (Holness et al. 2017a), with higher values found in the olivine-rich accumulation sequence on the floor. Holness et al. (2017a) attributed these localised high values to settling of the plagioclase component of the crystal cargo, with the buoyancy of plagioclase mitigated by it forming poly-mineralic clusters with olivine and spinel.

If we assume that the upwards-propagating solidification front in the Dun Raisburgh sill is grown entirely in situ, with no gravitationally-driven accumulation of the plagioclase component of the crystal cargo, a mechanically coherent framework can be formed with as little as 25 vol% plagioclase (Philpotts et al. 1998, 1999). Our chosen magma composition achieves this volume of plagioclase at 1105 °C, considerably earlier than augite saturation, consistent with the observed ophitic habit of the augite throughout the sill.

The olivine mode in the accumulation sequence on the floor of the fully solidified sill comprises not only the olivine cargo crystals but also an overgrowth on each grain and cluster formed during solidification of the carrier liquid. Using the olivine mode in the fully solidified rock, ϕ_obs_, and making assumptions about the porosity of the plagioclase-dominated mush into which the olivine settled, we can place constraints on the original volume of settled olivine crystals, ϕ_sed_, by subtracting the amount of olivine that grew from the interstitial liquid, ϕ_L_.

We estimated the maximum porosity of the mush to be 73 vol%, corresponding to a solid fraction comprising 25 vol% plagioclase (Philpotts et al. 1998, 1999) with 2 vol% olivine. In comparison with the interstitial liquid in the olivine accumulation on the floor of the Shiant Isles PCU, which had a composition close to that of the incoming carrier liquid, the interstitial liquid in the upwards-propagating plagioclase-dominated crystal mush on the floor of the Dun Raisburgh sill is more evolved since it has already crystallised plagioclase and some olivine to create the crystal mush. Therefore, the interstitial liquid composition in a mush comprising 25 vol% plagioclase (i.e. at 1105 °C) comprises SiO_2_: 46.5 wt%, Al_2_O_3_: 14.5 wt%, FeO: 14.9 wt%, MgO: 7.7 wt%, CaO: 13.7 wt%, Na_2_O: 2.6 wt%. Such a liquid can crystallise a maximum volume of 10 vol% olivine. Consequently, *ϕ*_sed_max_ = *ϕ*_obs_ – 10 × *ϕ*_L_max_.

The minimum porosity of the mush onto which the olivine grains and clusters settled corresponds to that at which the interstitial liquid becomes saturated in clinopyroxene. This occurs at 1095 °C, by which point the mush contains 67 vol% liquid of a composition SiO_2_: 46.4 wt%, Al_2_O_3_: 13.9 wt%, FeO: 15.6 wt%, MgO: 7.5 wt%, CaO: 13.9 wt%, Na_2_O: 2.58 wt%. Such a liquid can crystallise up to 6 vol% olivine. Consequently, *ϕ*_sed_min_ = *ϕ*_obs_ – 6 × *ϕ*_L_min_.

## Grain size measurement methods

### Average olivine grain size

The characterisation of particle size distribution is a prerequisite for understanding the fluid dynamic regime during solidification (Holness et al. 2017a). For polydisperse populations of spherical to sub-spherical particles, moment-based methods can be used to quantify three-dimensional crystal size populations from a two-dimensional thin section (Farr et al. 2017). For each sample, particle intersection diameters were obtained by measuring the outline of phenocrysts and polycrystalline mineral clusters drawn on thin section photographs. The area corresponding to the outline is calculated using ImageJ software (Schneider et al. 2012), and converted to the equivalent circular grain intersection {d_k_} (the Heywood diameter). We then used the method of Farr et al. (2017) to determine the equivalent 3D mean circle diameter at different stratigraphic heights in the olivine accumulation.

For each sample containing n particles of diameter {*d*_k_}, with 1 ≤ *k* ≤ *n*, the moment-based three-dimensional average diameter, d_i,j_, is defined by

$${d_{i,j}}={\left( {\frac{{\mathop \sum \nolimits_{k} d_{k}^{i}}}{{\mathop \sum \nolimits_{k} d_{k}^{j}}}} \right)^{\frac{1}{{~i - j}}}}$$

The d_i,j_ is used to determine the mean sphere diameter for a given sample

$${D_{3,2}}=\left( {\frac{{3\pi }}{8}} \right)~{d_{2,1}}~\left( {1~ \pm ~{e_{{\text{est}}}}} \right)$$

$${D_{4,3}}=\left( {\frac{{32}}{{9\pi }}} \right)~{d_{3,2}}~\left( {1~ \pm ~{e_{{\text{est}}}}} \right)$$

$${D_{3,1}}=\sqrt {1.5~{d_{2,0}}} ~\left( {1~ \pm ~{e_{{\text{est}}}}} \right)$$

where *D*_4,3_ and *D*_3,2_ are the volume- and surface-weighted mean diameters, respectively, and *D*_3,1_ is used to constrain particle accumulation rates. Estimates for the width of the distribution, ${\sigma _{{\text{est}}}}$, and the relative error in various mean diameters, *e*_*es*t_, can be derived from

$${\sigma _{{\text{est}}}}=\sqrt {{\text{ln}}\left( {\frac{{0.961{d_{3,2}}}}{{{d_{2,1}}}}} \right)} ~$$

$${e_{{\text{est}}}}=~\frac{{0.15}}{{\sqrt n }}{\text{exp}}\left( {5~{\sigma _{{\text{est}}}}} \right)$$

**Fig. 5:**
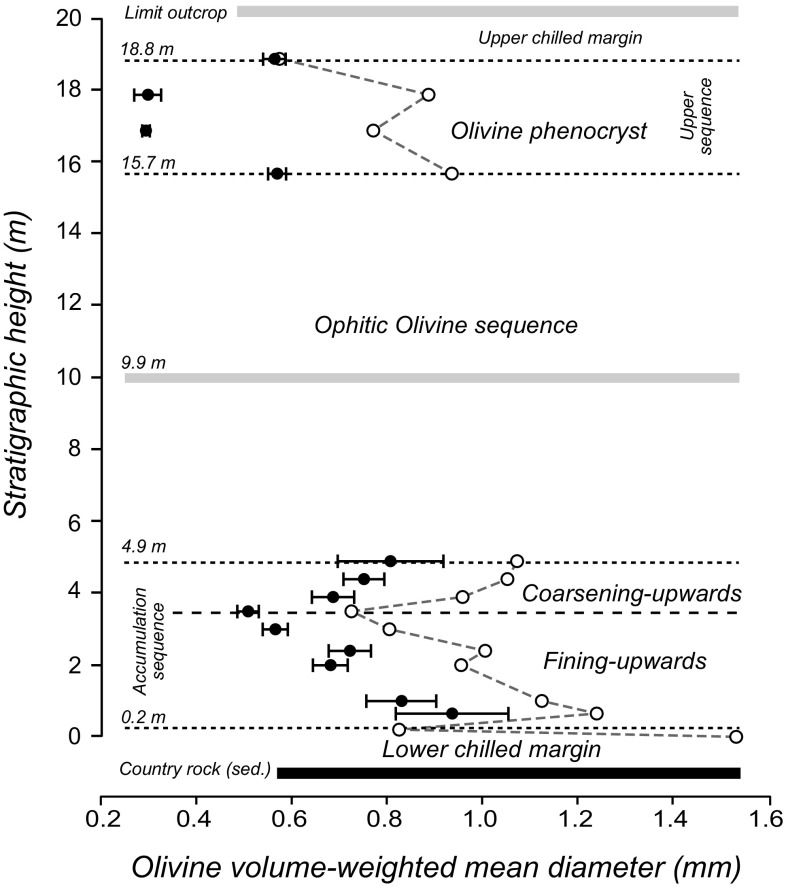
Variations in olivine grain size in the Dun Raisburgh sill. **a** Stratigraphic variation of the volume-weighted mean diameter of olivine clusters (i.e. including isolated grains). The open symbols are the uncorrected values of *D*_4,3_ and the black symbols are the corrected diameters accounting for post-accumulation overgrowth (D*_4,3_) (Farr et al. 2017). The horizontal dashed lines show the boundaries between the different units of the stratigraphic columns. Based on the olivine particle size profile, the accumulation sequence can be further subdivided into a fining-upwards sequence and an overlying coarsening-upwards sequence

The original grain size of the particles can be estimated by correcting the sizes observed in thin section from post-accumulation overgrowth, *δD*_*i,j*_, of the original crystal cargo during crystallisation of the carrier liquid:

$$\delta D=\frac{{{D_{3,2}}~\delta \varphi }}{{3{\varphi _{{\text{sed}}}}}}$$

where *δφ* is the volume fraction due to overgrowth (*δϕ* = *ϕ*_obs_ − *ϕ*_sed_). *ϕ*_obs_ in the accumulation sequence and the upper sequence are 11.5 vol% and 8.5 vol% respectively (Fig. 2). The average *ϕ*_sed_ is given by *ϕ*_sed =_ *ϕ*_obs_ – 0.8 × *ϕ*_L,_ with *ϕ*_L_ the average volume of interstitial liquid, 70 vol%. The results of these calculations are shown in Fig. 5.

### Plagioclase grain size

**Fig. 6:**
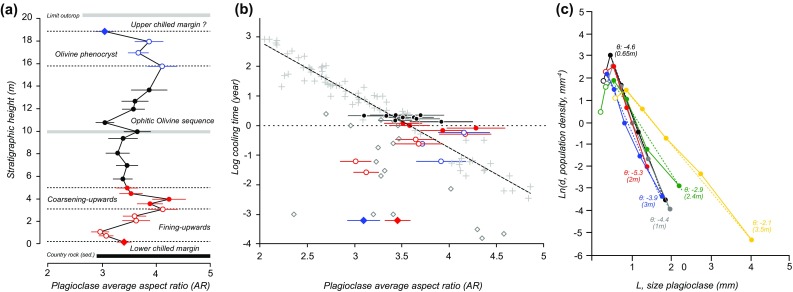
Variations in plagioclase shapeand grain size in the Dun Raisburgh sill. **a** Stratigraphic variation of average apparent aspect ratio of plagioclase, AR. **b** AR as a function of model calculated crystallization time (following Holness 2014). Circles: Dun Raisburgh sill; grey crosses and diamonds denote data from Holness (2014). The diamonds represent samples from the upper and lower marginal reversals. The samples from the centre of the Dun Raisburgh sill plot, within uncertainty, on the trend line defined by Holness (2014) (shown as a dashed line). **c** Plagioclase crystal size distributions in the fining-upwards samples, calculated following Marsh (1988). Linear regressions were calculated by removing the kinks at very low grain sizes (open symbols). Θ indicate the slope, [Gt]^−1^, for each sample. The numbers in brackets indicate the stratigraphic height of each sample of the fining-upwards sequence

Unlike olivine, plagioclase is tabular, so the moment-based method above cannot be used to quantify grain size. Furthermore, much of the plagioclase grew in situ in the sill, providing the opportunity to use grain size distributions to place constraints on crystallisation timescales. We therefore used the crystal size distribution approach of Marsh (1998) in which the population density of plagioclase, *n*, is given by

$$n={n_0}+\exp \left( {\frac{{ - L}}{{Gt}}} \right)$$

where *n*_0_ is the nucleation density, *L* the size of the plagioclase, *G* the growth rate (assumed constant) and *t* the residence time (Marsh 1998). Plagioclase long and short axes of a minimum of 150 grains per sample were measured using the software ImageJ (Schneider et al. 2012), and we used CSD correction software (Higgins 2000, 2002) to calculate the grain size distribution in samples from the lower 3.5 m of the sill. Using the method of Higgins (1994) we estimate that plagioclase grains form tablets with 3D aspect ratio 1:3:7 (samples RB12 and RB13), 1:4:7 (sample RB19), 1:3:3 (sample RB14 and RB15) and 1:3:10 (sample RB20). On the assumption that these samples contain grains of constant shape (which is unlikely to be entirely correct) we used these aspect ratios along with the population of long axes to calculate the CSD for each sample. The slope of a plot of *ln(n)* vs. *L* gives a measure of (*Gt*)^−1^. Results are shown in Fig. 6.

## Quantification of microstructural variations

### Sub-division of the accumulation sequence

A consideration of the stratigraphic variation of mineral modes and grain sizes of the olivine isolated grains and clusters in the Dun Raisburgh sill can be used to further sub-divide the sill (Fig. 5), with the sub-division of the accumulation sequence into two parts:

1. A lower fining-upwards sequence between 0.65 and 3.5 m stratigraphic height (including samples RB12 and RB20) is characterised by a reduction in olivine particle corrected mean diameter, D*_4,3_ (where D*_4,3_ = *D*_4,3_ − *δD*), from 0.9 ± 0.1 to 0.5 ± 0.02 mm (Fig. 5). The low olivine mode in this part of the intrusion points to accumulation of olivine occurring simultaneously with the growth on the sill floor of a crystal mush dominated by plagioclase. The average amount of post-accumulation overgrowth on settled olivine grains in this part of the stratigraphy, calculated as described earlier, *δD* = 0.27 ± 0.03, is constant.
2. A less well-developed coarsening-upwards sequence from 3.5 to 4.9 m stratigraphic height (between RB20 and RB27) that is characterised by a weak increase in average diameter of olivine clusters, D*_4,3_, from 0.7 ± 0.04 mm to 0.8 ± 0.1 mm (note that within the uncertainties, the average D*_4,3_ values do not vary, Fig. 5b). The average amount of post-accumulation overgrowth on settled olivine particles, clusters plus single grains, is the same as in the fining-upwards sequence, *δD* = 0.26 ± 0.02 mm.

The olivine grain size in the upper sequence (15.7 to 18.8 stratigraphic height) shows a poorly-defined downwards coarsening, with D*_4,3_ values ranging from 0.6 ± 0.03 to 0.2 ± 0.02 mm. The calculated amounts of overgrowth are relatively large in this part of the stratigraphy, δD ~ 0.5 mm, accounting for the difference between uncorrected and corrected diameter values.

### Plagioclase grain shape

Following Holness (2014), we also investigated the spatial variation of plagioclase grain shape, parameterised by the average apparent aspect ratio, AR, as viewed in thin section. The average apparent aspect ratio of plagioclase, AR, varies to form the M-shape characteristic of sills and lava lakes (Holness 2014), with lower AR in the slower cooled central parts of the sill, higher AR towards with margins, with well-defined marginal reversals (Fig. 6a) (data in Table 1). The presence of an upper marginal reversal in our sample suite suggests that our traverse ends close to the top of the sill, despite the lack of exposure of the upper contact.

We compared these results with those of Holness (2014) by using the same simple cooling model to calculate the crystallisation time. Accordingly, the time taken to crystallise each of our samples was calculated assuming conductive heat transfer, a crystallisation interval of 1200–1000 °C, constant release of latent heat of crystallisation, and a country rock temperature initially at 0 °C (Holness et al. 2012). The time taken to crystallise, *τ*, at any point in the sill, *x* (measured from the bottom chilled margin), is given by

$$\tau =0.1\frac{{{h^2}}}{\kappa }~\left[ {1+\cos \left( {\frac{{2\pi x}}{h}} \right)} \right],$$

where *h* is the sill thickness (assumed to be 18.8 m) and *κ* = 10^−6^ m^2^ s^−1^ is the thermal diffusivity. The plagioclase grain shape in the ophitic sequence (4.9–13.6 m stratigraphic height) is consistent with the relationship between solidification time and grain shape defined by Holness (2014) (Fig. 6b).

The plagioclase grain size data (Fig. 6c) show a homogeneous grain-size distribution in the lowermost 2 m of the intrusion, followed by a progressive increase in overall grain-size to 3.5 m stratigraphic height, although the small number of samples means that this progression is unlikely to be meaningful.

## Discussion

### Comparison of the Dun Raisburgh sill with the Shiant Isles Main Sill

**Fig. 7:**
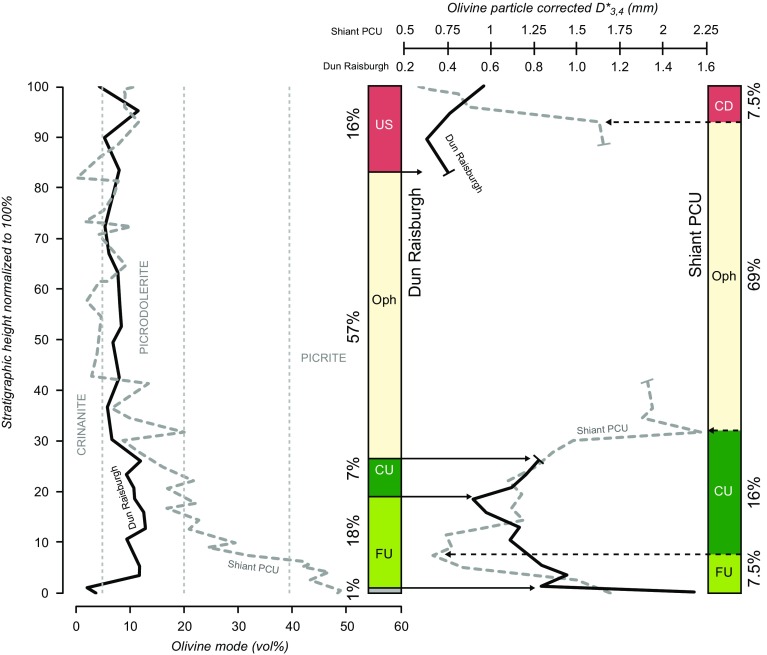
Comparison of the stratigraphic sequence from the Dun Raisburgh sill and the PCU from the Shiant Isles Main Sill (modified after Holness et al. 2017a) showing the olivine mode (vol%) together with the corrected volume-weighted mean diameter (D*_4,3_) of olivine particles. The plot of olivine mode is divided into lithologies according to the criteria of Gibson and Jones (1991) whereby crinanite < 5 vol%; picrodolerite 5–20 vol%; picrite 40–60 vol%. *FU* fining-upwards, *CU* coarsening-upwards, *Oph* ophitic sequence, *CD* coarsening-downwards (Shiant only), *UC* upper sequence (Dun Raisburgh only). Scale height: *H*_DR_ ~ 20 m; *H*_Shiant PCU_ ~ 135 m

Despite significant differences in mineral mode, the average olivine grain-size distribution in the Dun Raisburgh sill shows some similarities with that reported by Holness et al. (2017a) for the PCU of the Shiant Isles Main Sill (Fig. 7). Below, we discuss these similarities and differences and place constraints on the different processes responsible for the internal architecture of tabular intrusions.

#### Fining-upwards sequence

Both the PCU of the Shiant Isles Main Sill and the Dun Raisburgh sill display a well-defined fining-upwards sequence in the first 10 m and 3.5 m of the intrusion respectively (Fig. 7). In the Shiant Isles Main Sill PCU, the fining-upwards sequence has an average olivine mode of ~ 44 vol% and represents 7.5% (10.3 m) of the total 135 m thickness (Holness et al. 2017a) (Fig. 6), whereas the fining-upwards sequence in the Dun Raisburgh sill has an average olivine mode of ~ 11.5 vol% and represents 18% (3.5 m) of the total thickness (assumed to be 18.8 m): this, if scaled to the Shiant example, should not exceed 1.4 m in thickness. We attribute this difference to the nature of the crystal cargo. In the former, the crystal cargo is mainly composed of olivine phenocrysts with minor plagioclase and spinel (Gibb and Henderson 2006; Holness et al. 2017a), whereas in the latter the cargo was relatively rich in buoyant plagioclase (Fig. 4) which did not accumulate on the sill floor.

The nature of the floor on which the olivine of the crystal cargo settles will also influence observed particle size distribution in the fining-upwards sequence. Holness et al. (2017a) suggested that the accumulation sequence on the floor of the Shiant Isles PCU formed rapidly relative to solidification and was dominated by olivine, with an upwards-increasing component of plagioclase to maintain a mush porosity of ~ 55 vol%. Therefore, the thickness of the Shiant fining-upwards sequence was defined entirely by the accumulation of cargo crystals. In contrast, the accumulation sequence in the smaller and more rapidly cooled Dun Raisburgh sill comprises settled olivine crystals from the cargo, together with plagioclase grown in situ during simultaneous solidification, leading to a greater relative thickness of the fining-upwards sequence (Fig. 7).

The original volume of the crystal cargo in the carrier liquid can be calculated from the total amount of olivine sedimented on the sill floor, *ϕ*_sed_. In the lowermost 3.7 m of the stratigraphy (i.e. the fining-upwards part of the accumulation sequence, excluding the samples from the chilled margin), ϕ_sed_ is approximately constant at 5.3 ± 1 vol%. Therefore, we approximate the original volume of the crystal cargo by $\varphi _{{{\text{susp}}}}^{0}$ = (*ϕ*_sed_ × 3.7)/18.8, recalling that the total thickness of the sill is 18.8 m. We obtain an original bulk olivine cargo of 1.0 ± 0.2 vol%. This is smaller than the cargo carried by the Shiant PCU magma (~ 3.5 vol%, Holness et al. 2017a).

If a fining-upwards sequence results from hydrodynamic sorting during the deposition of a polydisperse population of dense crystals, there will be a correlation between the average particle size at any level of the stratigraphy of the cumulate pile and the volume of olivine remaining in suspension when the crystal load settles to form that portion of the pile.

The relationship linking the average volume fraction of sedimented olivine, *ϕ*_sed_(*x*), up to a stratigraphic height *x*, with the volume fraction *ϕ*_susp_(*x*) currently in suspension can be calculated thus: let the sill height be *h* and the original volume fraction of incoming crystal cargo ϕ^0^_susp_. The volume of this cargo over a unit area of the floor is therefore *h* $\varphi _{{{\text{susp}}}}^{0}$. Assuming no crystal growth during the sedimentation, once a depth, *x*, of sediment has accumulated, the volume of sedimented crystals over the unit area of the floor is *x. ϕ*_sed_(*x*), while the volume still in suspension is (*h-x*)*ϕ*_susp_(*x*). The sum of these must equal the original volume of the cargo, so:

$${\varphi _{susp}}\left( x \right)=\frac{{h*\varphi {0_{susp}} - x*{\varphi _{sed}}\left( x \right)}}{{h - x}}$$

**Fig. 8:**
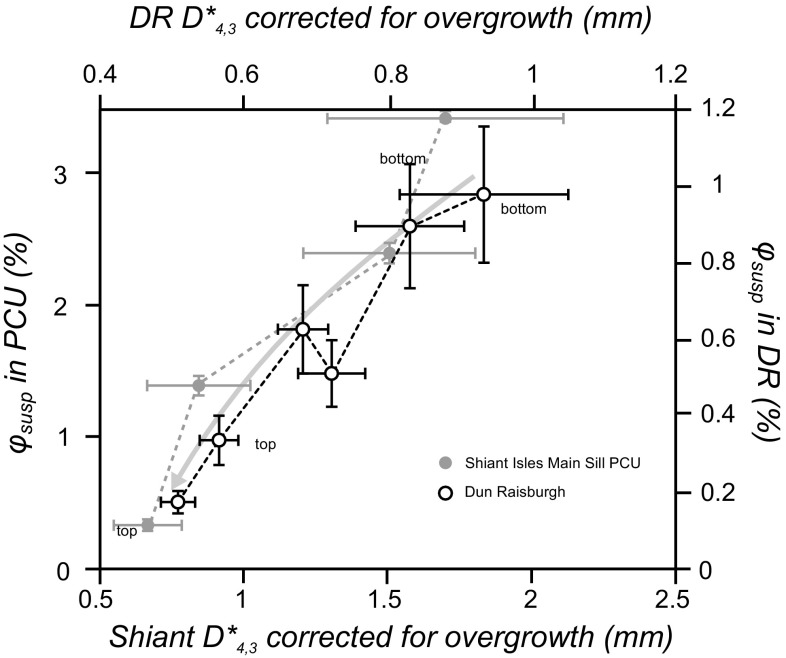
The calculated volume of olivine remaining in suspension at any moment (*ϕ*_susp_) as a function of the average diameter of olivine clusters (D*_4,3_) at the corresponding level in the fining-upwards sequence. The dashed lines represent the succession of samples from the bottom and the top of each fining-upwards sequence. The arrow highlights the general decrease in suspended grain size as the fining-upwards sequence builds up

We compared the *ϕ*_susp_ value to the corresponding corrected average diameter (D*_4,3_) as a function of stratigraphic position in the fining-upwards sequences of the Dun Raisburgh and the Shiant Isles PCU (Fig. 8). There is a general positive correlation between D*_4,3_ and *ϕ*_susp_ for both the Shiant Isles PCU and the Dun Raisburgh sill, consistent with the formation of the fining-upwards sequence by hydrodynamic sorting during settling.

**Fig. 9:**
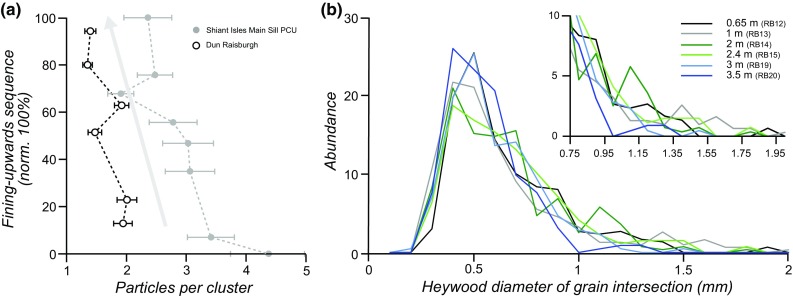
Grain and cluster size in the fining-upwards sequence. **a** Grains per cluster within the fining-upwards sequence (normalized to 100%) for the Dun Raisburgh sill and the Shiant Isles Main Sill PCU. Both profiles show a slight decrease in grains per cluster towards the top (arrow). **b** Size frequency of olivine clusters, quantified by the Heywood diameter of cluster intersections in thin section (all distribution are normalized to 100%). There is a gradual upwards loss of large clusters

The mechanisms responsible for the particle size distribution in the fining-upwards sequence can be distinguished using the results of analogue experiments examining grain aggregation by synneusis (Schwindinger 1999). Sedimentation from a static liquid in which grain collision is absent leads to a near-constant number of grains per cluster within the entire sequence. If grain amalgamation precedes sedimentation from a static magma and if, on average, grain size is constant regardless of the number of grains in a cluster, the number of grains per cluster will be at a maximum on the floor of the intrusion and will decrease exponentially towards one at the top of the fining-upwards sequence. Data from the Shiant Isles Main Sill PCU and the Dun Raisburgh sill show the average number of grains per cluster decreases only slowly through the fining-upwards sequence, consistent with the deposition of a pre-amalgamated crystal cargo in each case (Fig. 9a).

In an ideal scenario, particle size distributions in the fining-upwards sequence will be characterized by the progressive upwards phasing-out of each particle size class. This is seen both in the Shiant Isles Main Sill PCU, in which large grains progressively disappear towards the top of the fining-upwards sequence (Holness et al. 2017a), and also in the fining-upwards sequence of the Dun Raisburgh sill (Fig. 9b).

#### Coarsening sequences

Holness et al. (2017a) argued that the presence of a coarsening-upwards sequence in the Shiant Isles Main sill is direct evidence that the olivine settled from a convecting magma. Growth of the smallest olivine grains, remaining in suspension after the bulk of the cargo has settled, form clusters via synneusis or agglutination (Vance 1969; Schwindinger and Anderson 1989; Schwindinger 1999), with cluster size increasing with time. The progressive loss of these clusters from the convecting magma leads to the coarsening-upwards sequence. The presence of olivine phenocrysts in the top 10 m of the sill was explained by them having been brought to the top of the sill by convection currents where they became trapped in the downwards-propagating solidification front (Holness et al. 2017a).

In comparison with the Shiant Main Isles Sill, there is no strongly-developed coarsening-upwards sequence in the Dun Raisburgh sill: an upwards coarsening may perhaps be defined by 3–4 samples (Fig. 5a), although the average grain-size in the upper 3 samples is the same within uncertainty. However, the presence of a relatively thick upper sequence (16% of the total sill thickness—Fig. 7) containing olivine phenocrysts (Fig. 3i, j) at the roof of the Dun Raisburgh sill, could be temporally correlated with this weakly-defined upwards-coarsening sequence at the floor. This, combined with the uniform distribution of stellate plagioclase clusters throughout the sill, suggests that there is indeed a thin coarsening-upwards sequence in the Dun Raisburgh sill that might point to particle settling from a convecting magma in a similar way to that in the Shiant. However, the absence of an associated upwards increase in the number of olivine crystals per cluster (Fig. 2) in the putative coarsening-upwards sequence suggests that convection was limited in the Dun Raisburgh sill.

The difference in the strength of convection in these two intrusions must be a consequence of differences in their thickness and also in their immediate surrounding. The thinner Dun Raisburgh sill intruded cold host rock, with relatively rapid growth of plagioclase-rich solidification fronts, whereas the cooling rate in the thicker Shiant Isles Main Sill PCU was further reduced by the presence of an earlier, underlying, sill which was still hot at the time of PCU intrusion (Holness et al. 2017a and references therein). The presence of this additional heat source likely encouraged sustained vigorous convection.

### Sill solidification rate and olivine settling

The low olivine mode in the settled accumulation on the floor of the Dun Raisburgh sill demonstrates that the rate of upwards-propagation of the lower solidification front was commensurate with the olivine settling rate. The time required to form a plagioclase-dominated mush can be estimated from the preserved plagioclase size distribution (Marsh 1998; Jerram et al. 2003) (Fig. 5c) given an estimate of the growth rate of plagioclase, *G*. However, the plagioclase growth rate is not well known and is strongly dependent on the size of magma bodies: in slowly cooled magma chambers, *G* = 10^−8^ to 10^−10^ mm s^−1^, whereas in smaller bodies *G* = 10^−6^ to 10^−8^ mm s^−1^ (see review by Brugger and Hammer 2010). Growth rates were likely to be relatively fast in the 18 m thick Dun Raisburgh sill and, assuming constant growth rates of 10^−6^ to 10^−8^ mm s^−1^, and different aspect ratios for each stratigraphic heights (see earlier), the solidification front would have propagated upwards by 3.5 m (i.e. the thickness of the fining-upwards sequence) on timescales ranging from 2 weeks to 4 years. This is similar to a time of ~ 1 year estimated using the simple conductive heat transfer model of Holness et al. (2012) (see earlier).

**Fig. 10:**
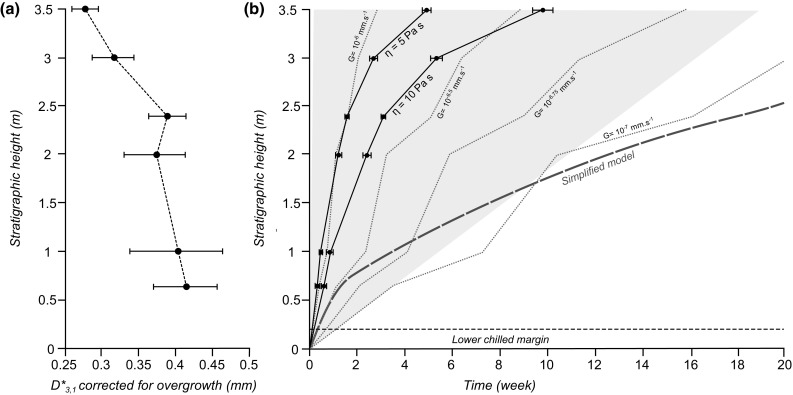
Crystallization of the fining-upwards sequence. **b** D*_3,1_ :corrected volume-weighted mean diameter *D*_3,1_ from overgrowth (*δD*_3,1_). **c** Comparison of the time required for formation of the fining-upwards sequence by sedimentation (heavy lines) with the time required for upwards-propagation of the solidification front (light lines), labelled with different assumed growth rates, *G*. For comparison, the time required for solidification according to the simplified relationship of Holness et al. (2012) is shown as a dashed line. The grey area shows the time required for the plagioclase clusters in the original crystal cargo to rise from the floor of the intrusion

Assuming Stokes settling of olivine single grains and clusters, where the settling velocity, *U*_*s*_, is given by

$${U_{\text{s}}}=~\frac{{g\Delta \rho }}{{18\eta }}{(D_{{3,1}}^{*})^2}$$

where *g* is the gravitational constant, *Δρ* the difference in density between the olivine particle (single grain or polycrystalline cluster) and the magma, *η* is the magma viscosity, and D*_3,1_ is the mean diameter *D*_3,1_ corrected for any post-accumulation overgrowth, which varies with stratigraphic height (Fig. 10a). We can calculate the rate at which the layer, of thickness *H*, accumulates by relating the settling of crystals to the thickness of the evolving cumulate pile following Farr et al. (2017):

$$\dot {H}=~\frac{{{\varphi _{{\text{susp}}}}}}{{{\varphi _{{\text{sed}}}}}}~{U_{\text{s}}},$$

where *Ḣ* is the growth rate of the layer, *U*_s_ is the Stokes settling velocity, *ϕ*_susp_ is the volume fraction of olivine in the incoming magma and ϕ_sed_ the is the volume of olivine settled on the floor of the intrusion. We set *Δρ* = 560 kg m^− 3^ (based on our thermodynamic modelling of magma at *T* = 1100 °C) and *η* = 5–10 Pa s (Gibb and Henderson 2006). The time taken for Stokes settling from a given stratigraphic height, *x*_*n*_, within a sill of overall height, *h*, is *t* = *(h−x*_*n*_*)*/*Ḣ*. Step-wise integration of the time required to form the ~ 3.5 m thick fining-upwards sequence indicates a time-scale of 5–10 weeks, commensurate with that of the upwards-propagation of the solidification front (Fig. 10b).

Reconciliation of these two different sets of calculations can be attained for values of growth rate between 10^−6.0^ and 10^−6.75^ mm s^−1^, for which the timescale for formation of a 3.5 m thick solidification front (3–15 weeks) is within error of the time required for olivine settling (5–10 weeks) (Fig. 10b). These values of growth rates are consistent with that usually chosen for crystals growing in ascending hydrous magma, ~ 10^−7^ mm s^−1^ (e.g. Mastrolorenzo and Pappalardo, 2006; Salisbury et al., 2008).

To investigate how the behaviour of the plagioclase component of the crystal cargo might have affected olivine settling, we calculated the time during which plagioclase clusters would rise from the floor of the intrusion to a height corresponding to the top of the fining-upwards sequence (3.5 m). We used an average *Δρ* of 76 kg m^−3^ (Fig. 4b) and an average cluster size of 0.25–0.3 mm, as observed in the chilled margin. Clusters in this size range float to heights above the fining-upwards sequence on timescales between 1 day and 20 weeks (Fig. 10b), within error of the olivine cargo settling time. Consequently, it is unlikely that the behaviour of buoyant cargo plagioclase would have disturbed the formation of the accumulation sequence.

## Conclusions

Petrographic observations and grain size-distributions in the Dun Raisburgh sill confirm and refine the hypothesis of Holness et al. (2017a) that the spatial variation of grain size in a polydisperse settled accumulation can be used to constrain the mechanisms responsible for the internal architecture of tabular intrusions.

Despite being of smaller size (~ 20 m thick), the Dun Raisburgh sill has a similar stratigraphic sequence to that of the PCU of the the Shiant Isles Main Sill (135 m thick) and was formed by a magma with a broadly similar crystal cargo comprising ~ 3 vol% phenocrysts of both olivine and plagioclase. The internal structure of both sills can be explained by an early post-emplacement period during which the biggest olivine grains settled to form a fining-upwards sequence on the sill floor, while the smallest particles remained in suspension. The Dun Raisburgh crystal cargo additionally contained significant amounts of buoyant plagioclase that concentrated in the centre of the intrusion. In contrast to the Shiant Isles Main Sill, convection was limited in the thinner Dun Raisburgh sill, resulting in a poorly-defined and thin coarsening-upwards sequence. Microstructural observations combined with thermodynamic modelling and CSD calculations indicate that the rapid growth of a rigid, porous (~ 70% interstitial liquid) plagioclase-dominated mush created an upwards-propagating solidification front at a commensurate rate as the settling of the olivine crystal cargo.
